# Kundalini Yoga for Post-Treatment Lyme Disease: A Preliminary Randomized Study

**DOI:** 10.3390/healthcare10071314

**Published:** 2022-07-15

**Authors:** Lilly Murray, Charles Alexander, Clair Bennett, Mara Kuvaldina, Gurucharan Khalsa, Brian Fallon

**Affiliations:** 1 Lyme & Tick-Borne Diseases Research Center, Department of Psychiatry, Columbia University Irving Medical Center, New York, NY 10032, USA; cb3492@cumc.columbia.edu (C.B.); mk4480@cumc.columbia.edu (M.K.); 2 New York State Psychiatric Institute, New York, NY 10032, USA; 3 Private Practice, Southport, CT 06890, USA; chipalex13@aol.com; 4 Fish Interfaith Center, Chapman University, Orange, CA 92866, USA; khalsa@chapman.edu

**Keywords:** post-treatment Lyme disease syndrome, Kundalini yoga, meditation, symptom burden, cognitive functioning

## Abstract

This study examined the adherence to and the potential benefit of Kundalini yoga (KY) for post-treatment Lyme disease syndrome (PTLDS). Participants were randomly assigned to 8 weeks of a KY small-group intervention or a waitlist control (WLC). Adherence was measured as attendance at KY group sessions. Primary outcomes assessed pain, pain interference, fatigue, and global health. Secondary outcomes assessed multisystem symptom burden, mood, sleep, physical and social functioning, cognition, and mindfulness. Linear mixed models were used to test changes in outcomes over time as a function of group assignment; intercepts for participants were modeled as random effects. Although the target sample size was 40 participants, the study concluded with 29 participants due to recruitment challenges. No KY participants dropped out of the study, and participants attended 75% of group sessions on average, but WLC retention was poor (57%). Regarding primary outcomes, there was no significant interaction between group and time. Regarding secondary outcomes, there was a significant interaction between group and time for multisystem symptom burden (*p* < 0.05) and cognition (*p* < 0.01); KY participants reported improved multisystem symptom burden and cognition over the course of the study compared to WLC participants. To enhance recruitment and retention, future trials may consider expanding geographic access and including supportive procedures for WLC participants. This preliminary study supports the need for a larger study to determine if KY reduces multisystem symptom burden and enhances cognition among people with PTLDS.

## 1. Introduction

Lyme disease is the most common vector-borne illness in the United States, with increasing prevalence and geographic spread [1]. Although prognosis is favorable following uncomplicated infection and appropriate antibiotic therapy [2], approximately 10–20% of patients experience persistent fatigue, musculoskeletal pain, cognitive difficulties, or other symptoms for six months or longer after treatment [3]. This is referred to as post-treatment Lyme disease syndrome (PTLDS) [2]. Possible explanations for PTLDS include a persistent infection not responsive to antibiotic therapy or post-infectious processes, such as an abnormally activated immune response [2] or central sensitization [4]. Although the Infectious Disease Society of America (IDSA) discourages extended antibiotic treatment for PTLDS [5], many patients do receive additional courses of antibiotics, with mixed clinical outcomes [6]. Safe, effective, and accessible treatment alternatives for people with PTLDS are urgently needed.

Yoga and meditation, often practiced together, involve one or more combinations of the basic elements of postures, contemplation, attention, and directed breathing [7,8,9]. These heterogenous contemplative practices have gained interest as adjunctive treatments for a range of chronic conditions [9,10,11,12,13,14,15,16,17,18,19,20,21,22,23,24,25,26,27,28]. Potential benefits of contemplative practices may be mediated by several pathways. One proposed mechanism relates to effects on the autonomic nervous system attenuating the stress response [29,30]. Stress is a common experience in chronic disease [31], and is widely associated with fatigue and pain [32,33]. Another potential mechanism is the modulation of the immune response; meditation has been shown to attenuate pro-inflammatory processes, enhance cell-mediated immunity, and slow cellular aging [16,34,35,36,37]. Contemplative practices have also been reported to reduce the psychosocial and existential distress associated with chronic disease [26]. This may occur through the cultivation of equanimous self-awareness, leading to a new mindset about one’s body and experience of illness [9].

The efficacy of contemplative practices for PTLDS has not yet been investigated. However, yoga and meditation have been shown to alleviate fatigue, pain, sleep disturbance, psychological distress, anxiety, and cognitive impairment—common symptoms of PTLDS [38]—in patients with other chronic conditions [8,13,14,15,16,17,24,26,28,39,40]. Thus, we hypothesized that contemplative practice may similarly benefit people with PTLDS. 

Kundalini yoga (KY) is a contemplative practice that integrates movement, breathwork, and meditation [41]. This combination may enhance immune function [10] and buffer the autonomic stress response [30]. Research to date on KY for chronic conditions is limited, but generally favorable. A study of adults with mild cognitive impairment reported that KY and memory enhancement training conferred similar cognitive benefits [39]. A study of adults with obsessive-compulsive disorder (OCD) showed that KY improved OCD symptoms, mood states, anxiety, and depression—but did not improve functional health—relative to relaxation response meditation [10]. KY, relative to stress education, was shown to be effective for generalized anxiety disorder, but to a lesser extent than gold-standard cognitive behavioral therapy [21]. As an added benefit, KY was associated with fewer adverse events than other yoga styles in one cross-sectional survey [42].

This preliminary study examined the adherence to and potential benefit of an 8-week KY intervention among small groups of patients with persistent pain or fatigue after treated Lyme disease. We defined treatment adherence as participant attendance at weekly KY group trainings. Since pain and fatigue are ubiquitous among people with PTLDS, we focused on these symptoms as primary outcome measures, as well as perceived global health. Secondary outcome measures were functional impairment, multisystem symptom burden, depression, anxiety, sleep disturbance, physical functioning, social functioning, cognition, and mindfulness. We also assessed intolerance of uncertainty, pain catastrophizing, lifetime traumatic events, stress over the past month, health anxiety, and perception of illness timeline as pre-treatment variables to assess impact on outcomes.

## 2. Materials and Methods

### 2.1. Ethical Approval and Registration

This study was approved by the New York State Psychiatric Institute Institutional Review Board at Columbia University Irving Medical Center (#6927) and registered at ClinicalTrials.gov (accessed on 28 May 2022) (“Meditation and Stretching for Post Treatment Lyme Disease Syndrome,” NCT02344537).

### 2.2. Participants

Our target sample size for this study was 40 adults. This sample size was selected to provide preliminary data on adherence to and potential benefit of the KY intervention. Research assistants randomly assigned participants to a KY intervention group or to a waitlist control (WLC) group using an online random number generator in cohorts of 8 to 12 people at a 1:1 KY:WLC distribution. A KY intervention group size of 4 to 6 people was selected due to the small size of the office, and to minimize delay between enrollment and start of treatment. When one cohort formed of 8 to 12 participants was to be randomized to the KY or WLC group, recruitment began for the next cohort. Participants randomized to the WLC group were offered KY training at no cost after 8 weeks. 

Participants were recruited using a Columbia University Irving Medical Center research recruitment website, the Lyme & Tick-Borne Diseases Research Center website and Facebook page, and flyers posted at local physician offices between January 2015 and December 2018. Research assistants conducted telephone screenings to assess eligibility. People above 18 years of age were invited to participate in the study if they (1) had a clinician diagnosis of Lyme disease at least 6 months prior to assessment, (2) received antibiotic treatment for Lyme disease that met or exceeded the duration recommended by the IDSA, (3) reported current symptoms that started within six months of the Lyme disease diagnosis and persisted for at least 6 months, and (4) had a primary complaint of pain or fatigue that met severity criteria: a score of 4 or higher out of 10 on a pain visual analog scale or a t-score of 60 or higher on the 7-item PROMIS Fatigue Short Form. Exclusion criteria were (1) severe depression, (2) physical disability or medical illness that might make participation difficult, (3) another medical or psychiatric illness that might better account for current pain or fatigue than PTLDS, and (4) prior lifetime practice of at least one month of daily KY or current daily practice of any yoga or meditation.

### 2.3. Measures

#### 2.3.1. Adherence

Adherence to the KY intervention was assessed by taking attendance at weekly group sessions. Participants were also asked to maintain a daily log on paper documenting time spent and open-ended reflections on their independent KY practice.

#### 2.3.2. Primary Outcome Measures

Primary outcomes were assessed at baseline, week 4, and week 8 of the study. Pain over the past two weeks was assessed using a 10-point visual analog scale. Pain interference over the past week was assessed using the 6-item PROMIS Pain Interference v.1.0 [43], in which a higher score indicates more pain interference with activities of daily living. Fatigue over the past week was assessed using the 7-item PROMIS Fatigue Short Form 7a v1.0, in which a higher score indicates more fatigue [44]. Perceived global health was assessed using a single question rated on a 5-point scale, in which a higher score indicates better perceived global health [45,46].

#### 2.3.3. Secondary Outcome Measures

Secondary outcomes were assessed at baseline, week 4, and week 8 of the study. Multisystem symptom burden over the past two weeks was assessed using the General Symptom Questionnaire-30 [47], in which a higher score indicates higher multisystem symptom burden. Depression, anxiety, sleep disturbance, physical function, and social activities over the past week were assessed using PROMIS measures, in which higher scores indicate more of the construct measured: Depression Short Form 6A v.1.0 [48]; Anxiety Short Form 6A v.1.0 [48]; Sleep Disturbance Short Form 6A v.1.0 [49]; Physical Function Short Form 4a v1.0 [50]; and Ability to Participate in Social Roles and Activities Short Form 8a v2.0 [51]. In addition to the PROMIS Depression Short Form 6A, depression was also assessed using the Beck Depression Inventory (BDI) [52], in which a higher score indicates more severe depression. Cognition over the past week was assessed using the NeuroQoL Applied Cognition General Concerns Short Form v1.0 [53], in which a higher score indicates fewer cognitive concerns. Mindfulness was assessed using the Mindful Attention Scale [54], in which a higher score indicates greater mindfulness.

#### 2.3.4. Pre-Treatment Variables

Pre-treatment variables were assessed once, after completing informed consent. Intolerance of uncertainty was assessed using the prospective subscale from the Intolerance of Uncertainty Scale Short Form [55]. Pain catastrophizing was assessed using the catastrophizing subscale of the Coping Strategies Questionnaire [56]. Lifetime traumatic events were assessed by summing the number of high-magnitude stressors reported in the Trauma History Screen [57]. Stress over the past month was assessed using the Perceived Stress Scale 4 Short Form [58]. Health anxiety was assessed using the Whitley Index [59]. Perception of illness timeline was assessed using a single item (“How long do you think your illness will continue?”) from the Brief Illness Perception Questionnaire [60]. This item was selected because perception of illness timeline was believed to be particularly relevant to treatment outcomes. 

### 2.4. Intervention

When a cohort of 8 to 12 eligible adults were identified, they were invited to complete informed consent and pre-treatment measures. Participants were subsequently randomized to the KY or WLC group.

Participants assigned to the KY intervention met in groups of 4 to 6 people once per week for 8 weeks to complete 1.5-h sessions of KY practice. The meetings were led by one of the study investigators (CA), a certified KY instructor with over 500 h of training, in Southport, CT. Attendance was taken at each session. Participants were also asked to complete and document duration of independent practice each day.

During the first of the 8 weekly group sessions, participants were trained to complete all aspects of the KY protocol, which consisted of light stretching with rhythmical movements, directed breathing, and guided meditation (described in Appendix A). Each participant received a paper copy of the stretching exercises, a guided meditation CD or MP3, and a paper daily practice log. Participants were encouraged to complete all aspects of the KY protocol (light stretching, directed breathing, and guided meditation) and complete the practice log at home every day. The complete daily practice routine was approximately 35 min. During subsequent weekly group sessions (weeks 2–8), participants completed the entire KY routine together and discussed their experiences while practicing at home. Participants in the KY and WLC groups completed primary and secondary outcome measures at baseline, week 4, and week 8, as described above.

Participants did not receive financial compensation for this study.

### 2.5. Statistical Analysis

To assess adherence to the small-group intervention, we counted the number of KY group sessions attended by each participant over the 8 weeks. To assess adherence to independent home practice, we counted the number of hours of daily practice reported on the paper logs by each participant over the 8 weeks.

To assess change in primary and secondary outcome measures over the 8 weeks, we used linear mixed models (LMMs) to test fixed and random effects with restricted maximum likelihood estimation. These analyses were conducted using R version 3.5.3 [61], with packages lme4 [62] and lmerTest [63] for mixed-effects models. Models were initially fitted with group (KY, WLC) as the between-subjects factor, time (baseline, week 4, and week 8) as the within-subjects factor, and interaction between group and time as a fixed effect. Intercepts for every participant were modeled as random effects. As a second step, the effect of cohort was modelled as a random effect in all models to account for the nested structure of the study design. In all models, cohort contributed 0 or negligible additional variance to the model. Comparison of Akaike information criteria (AIC) values between the simple and more complex structure indicated that the simple structure resulted in better model fit for all outcome variables. Consequently, cohort was not included as a random effect in any of the final models. All degrees of freedom were calculated with Satterthwaite approximation. 

Visual inspection of residual plots did not reveal substantial deviations from normality or homogeneity of variances. Models were run with and without outliers. Since the presence of outliers did not substantially alter results for any of the models, outliers were retained in the final analyses. LMMs were conducted according to a per protocol approach, with no imputation for missing data. One participant in the KY group began a course of antibiotics during the study; only data collected prior to the antibiotic treatment were included for this participant. As the main parameters of interest were interaction effects between group and time for all outcome variables, these are reported in text where significant. 

To assess whether pre-treatment variables were related to outcomes, mean change scores were calculated for primary and secondary outcome measures from baseline to week 8 for the KY group. Missing data were handled with pairwise deletion (*n*s ranged from 9 to 13). Due to small sample sizes, spearman rank correlations were conducted to assess associations between pre-treatment variables and change scores.

## 3. Results

**Figure 1:**
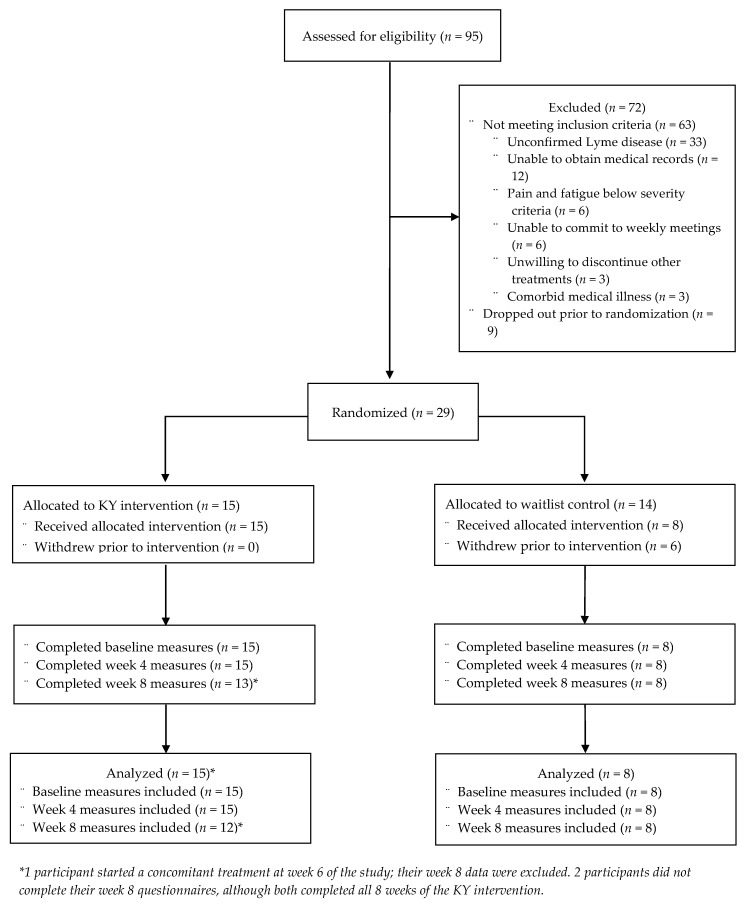
CONSORT flow diagram.

The participant flow diagram for this study is shown in Figure 1. A total of 32 of the 95 screened individuals (33.7%) were eligible for the study. More than half of the individuals screened were ineligible due to unconfirmed Lyme disease diagnosis. Of the 32 eligible participants, 9 dropped out prior to randomization, and 29 enrolled. 

Participant demographic and clinical characteristics are shown in Table 1. Our sample consisted primarily of white, middle-aged participants. Of note, WLC participants were significantly older than KY participants (F_1,21_ = 5.72, *p* = 0.03). No other demographic differences were observed between groups.

Due to recruitment difficulties, we concluded the study with 29 rather than 40 participants. Three cohorts of participants were formed. Across the three cohorts, 15 participants were assigned to the KY group, and 14 were assigned to the WLC group. No participants assigned to the KY group dropped out, although two did not complete their week 8 questionnaires despite continued involvement in weekly meetings and correspondence. Six participants assigned to the WLC group dropped out of the study after randomization but prior to completing baseline outcome measures. Therefore, our dataset for analysis comprised 15 KY participants and 8 WLC participants. 

When prompted to share KY practice experiences at each weekly meeting, no KY participants reported adverse events during independent or group practice.

### 3.1. Adherence

The proportion of weekly group sessions attended, averaged across KY participants, was 6 out of 8 (75%). Three out of 15 participants (20%) attended all 8 weekly sessions. None of the participants attended less than 4 out of 8 sessions. One participant in the KY group began antibiotic treatment during week 5 of the study; although their subsequent self-report measures were excluded from analyses, the participant continued to practice KY daily and attended subsequent group sessions. 

We were unable to determine adherence to daily KY practice due to the incomplete use of daily logs by many participants, and lack of return of daily logs by three participants.

### 3.2. Primary Outcome Measures

LMM analyses revealed no significant interaction effects of group and time for the primary outcome variables of pain, pain interference, or global health. There was a trend towards significance for the primary outcome of fatigue (F_1, 37.41_ = 3.60, *p* = 0.07). See Table 2 for means and standard deviations (SD) of all outcome variables by group at baseline, week 4, and week 8. 

### 3.3. Secondary Outcome Measures

LMM analyses revealed a significant interaction effect between group and time for multisystem symptom burden (F_1, 31.14_ = 5.58, *p* < 0.05) and cognition (F_1, 40.01_ = 11.51, *p* < 0.01). The KY group reported a significant reduction in multisystem symptom burden and cognitive concerns compared to the WLC group over the course of the study. There were no significant interaction effects for group and time for any of the other secondary outcome variables. 

### 3.4. Pre-Treatment Variables

Intolerance of uncertainty was positively correlated with fatigue change scores (r_s_ = 0.73, *p* = 0.01; *n* = 12, 95% CI(0.46, 0.98)), multisystem symptom change scores (r_s_ = 0.67; *p* = 0.05, *n* = 9, 95% CI(0.02, 0.92)), and depression, as measured by the BDI change scores (r_s_ = 0.67, *p* = 0.03, *n* = 10, 95% CI(0.07, 0.92)). Health anxiety was positively correlated with fatigue change scores (r_s_ = 0.75, *p* = 0.01, *n* = 10, 95% CI(0.24, 0.94)), BDI change scores (r_s_ = 0.80, *p* = 0.02, *n* = 8, 95% CI(0.19, 0.96)), and multisystem symptom change scores (r_s_ = 0.90, *p* = 0.01, *n* = 7, 95% CI(0.46, 0.99)). Given that no other correlations were significant, no additional analyses were conducted.

## 4. Discussion

This is the first preliminary study to explore the adherence to and potential benefit of KY for people with persistent pain or fatigue after treated Lyme disease. Retention was excellent in the KY group (100%), but poor in the WLC group, with 42% of WLC participants dropping out after learning of their randomization assignment. No adverse events were reported during the trial. Adherence to group sessions was favorable among our KY participants. While there were no group differences in the primary outcomes, KY was associated with improvement on two of the secondary outcomes: multisystem symptom burden and cognition. To inform future trials, we discuss these findings below.

Regarding recruitment, we were unable to achieve our goal of 40 participants despite conducting our study in a suburban community with a high incidence of Lyme disease. Ultimately, rather than prolonging the study enrollment period, we opted to stop at 29 participants, with 23 who completed the full trial. It seemed unlikely that we could increase our sample size without a lengthy and expensive advertising effort. We found that PTLDS patients expressed substantially less interest in our KY study than our pharmacologic and diagnostic studies. We suspect one contributing factor was the ubiquity of other opportunities to practice yoga and meditation in the Southport, CT area online, or in fitness studios. Moreover, many patients expressed skepticism that KY could help alleviate their symptoms. Others explained that clinicians have attributed their illness to psychiatric or functional etiologies, a common experience of people with PTLDS [3]; these patients were wary to try KY as this could be seen as an implicit acknowledgement that “it’s all in my head.” These barriers illustrate the pervasiveness of mind–body dualism in Western culture and medicine, limiting acceptance of potentially therapeutic interventions and disempowering patients [64]. 

Regarding adherence and retention in the KY group, participants attended an average of 75% of the weekly group sessions. No KY participants dropped out. Adherence in other yoga studies varies widely, but this finding is generally consistent with similar trials [12,26,29,30]. Due to poor compliance with daily practice documentation, we were unable to report how often participants practiced KY independently. 

Regarding adherence and retention in the WLC group, 43% of participants dropped out once they learned of their randomization to the WLC group. All WLC participants who completed post-randomization baseline assessments stayed in the trial. Since yoga and meditation protocols are easily accessible elsewhere, participants who dropped out of the WLC group may have opted for more convenient practice opportunities outside of the study. 

Regarding primary outcome measures, pain, pain interference, fatigue, and global health were not significantly improved in the KY group compared to the WLC group despite research supporting the benefits of yoga and meditation in other chronic conditions [8,13,14,15,16,17,26]. The small sample size in this study may have reduced sensitivity to detect effects. It is also possible that sustained practice beyond 8 weeks [13,25,65] or more frequent group sessions [27] are needed to achieve these benefits from KY. Moreover, KY’s broad focus on visualization and acceptance may facilitate pain coping [13] more than pain reduction, the former of which was not measured in this study. 

Regarding secondary outcome measures, multisystem symptom burden as assessed by the GSQ-30 was significantly improved in the KY group compared to the WLC group. This is notable as our study sample was heterogeneous: some had marked fatigue, some had marked pain, and some had both. However, the improvement in multisystem symptom burden may seem to conflict with the lack of improvement on the global health primary outcome measure. It is possible that the GSQ-30 is more sensitive in capturing overall health improvements than the single-item global health rating, especially since the former was specifically developed to assess common symptoms of Lyme disease. These data suggest that the GSQ-30 should be considered in future PTLDS studies as a primary outcome to assess treatment response. While a prior validation study confirmed the sensitivity of the GSQ-30 to detect change among patients with acute Lyme disease [47], this study suggests the GSQ-30 may also be sensitive in detecting change among patients persistent symptoms after treated Lyme disease.

Significant improvement was also noted on the secondary outcome measure of cognition. The NeuroQoL Cognitive General Concerns questionnaire assesses many common complaints among people with PTLDS, such as memory, attention, multi-tasking, and processing speed; patients often use the phrase “brain fog” to describe these symptoms [38]. While self-report cognitive measures do not necessarily correlate with objective neurocognitive assessments [65], prior research suggests that KY may confer objective cognitive benefits. In one study of adults with mild cognitive impairment, KY led to improvements in memory and executive functioning on formal neurocognitive testing [39]. A review of the impact of meditation (a core component of KY) on cognition in older adults concluded that meditation can positively affect attention, memory, verbal fluency, and cognitive flexibility [20]. Whether the attenuated cognitive concerns reported in our KY group are due to enhanced brain function or feeling better overall, as suggested by reduced multisystem symptom burden, is unclear. 

Regarding pre-treatment variables, participants who reported a higher intolerance of uncertainty experienced a greater improvement in fatigue, multisystem symptom burden, and depression, as measured by the BDI, than participants who reported less intolerance of uncertainty. Participants with a higher health anxiety also experienced a greater improvement in fatigue, multisystem symptom burden, and depression, as measured by the BDI, than participants with a lower health anxiety. It is possible that intolerance of uncertainty and health anxiety predict treatment success. However, it is important to note that our small sample size entails a high likelihood of inflated correlation coefficients, further evidenced by wide confidence intervals.

The primary strength of this randomized controlled preliminary trial is its concept: this is the first study to explore a behavioral intervention for PTLDS. PTLDS treatment research to date has focused largely on antibiotic therapy, with mixed results. Some small studies have reported benefits, but other larger studies have reported no benefits [6]. While persistent infection is one potential cause of PTLDS, post-infectious mechanisms may also play a role [66]. Alternative treatment options are needed for these patients. The possibility that a safe and accessible intervention such as KY could lead to reduced multisystem symptom burden and improved cognitive functioning deserves further investigation. An additional strength of this study is that it was conducted in a Lyme-endemic community setting by an experienced physician and KY teacher. 

The primary limitation of this study is the small sample size, which limits generalizability and confidence in our results. An additional limitation is that we did not include a long-term follow-up assessment. Although we initially planned a 6-month follow-up, we ultimately reduced our focus to the 8-week primary endpoint. 

This study leads to several recommendations for future trials of yoga and meditation for PTLDS. To enhance enrollment, future trials should consider using a network of multiple sites, targeting large metropolitan areas, offering financial compensation to participants, and employing a virtual platform to expand geographic access to the study. To enhance data collection, a more accessible and reliable platform than paper (e.g., a smartphone app) should be offered for participants to submit daily practice logs. To enhance conceptual rigor, future trials may consider including a control group that addresses the beneficial effects of social interaction in weekly meetings—for example, a Lyme disease support group. This might also enhance control group engagement, thereby reducing dropouts. 

## 5. Conclusions

Supplementary treatment modalities for PTLDS are urgently needed given the increasing prevalence of Lyme disease. KY is safe, accessible, and cost-effective. Excellent retention among our KY participants suggests that the group therapy format was effectively engaging. Moreover, our preliminary data suggest that KY is potentially effective in reducing some of the core symptoms of PTLDS. Although we encountered significant recruitment challenges, this preliminary trial yielded valuable insights for future studies. The efficacy of KY for PTLDS warrants evaluation in a larger randomized trial informed by these insights. 

## Figures and Tables

**Table 1:** Demographics and clinical characteristics of participants who received allocated intervention.

	KY	WLC	Overall
	(*n* = 15)	(*n* = 8)	(*n* = 23)
Sex = Male (*n* (%))	7 (46.7)	4 (50.0)	11 (47.8)
Ethnicity (*n* (%))			
Hispanic	1 (6.7)	0 (0.0)	1 (4.3)
White (non-Hispanic)	12 (80.0)	6 (75.0)	18 (78.3)
Not reported	2 (13.3)	2 (25.0)	4 (17.4)
Age (M (SD)) *	48.47 (12.99)	62.62 (14.51)	53.39 (14.90)
Marital Status (*n* (%))			
Single (never married)	1 (6.7)	2 (25.0)	3 (13.0)
Married/Living with partner	11 (73.3)	5 (62.5)	16 (69.6)
Divorced/Separated	1 (6.7)	0 (0.0)	1 (4.3)
Not reported	2 (13.3)	1 (12.5)	3 (13.0)
Education (*n* (%))			
Completed high school or GED	2 (13.3)	0 (0.0)	2 (8.7)
Some college (less than 4 years)	4 (26.7)	1 (12.5)	5 (21.7)
Completed 4-year college degree	2 (13.3)	3 (37.5)	5 (21.7)
Completed post-college degree	4 (26.7)	2 (25.0)	6 (26.1)
Not reported	3 (20.0)	2 (25.0)	5 (21.7)
Employment (*n* (%))			
Employed at least half-time for pay	1 (6.7)	0 (0.0)	1 (4.3)
Employed full-time for pay	2 (13.3)	4 (50.0)	6 (40.0)
Unemployed more than 6 months	4 (26.7)	0 (0.0)	4 (17.4)
Disabled	4 (26.7)	0 (0.0)	4 (17.4)
Retired	0 (0.0)	1 (12.5)	1 (4.3)
Other	1 (6.7)	2 (25.0)	3 (13.0)
Not reported	3 (20.0)	1 (12.5)	4 (17.4)
Medical Utilization (*n* (%)) ^a^			
Lifetime: medical hospitalization	11 (78.6)	6 (75.0)	17 (77.3)
Lifetime: psychiatric hospitalization	3 (21.4)	0 (0.0)	3 (13.6)
Lifetime: major surgery	11 (78.6)	6 (75.0)	17 (77.3)
Past 3 months: emergency room visit	1 (7.1)	0 (0.0)	1 (4.5)
Past 3 months: urgent care visit	2 (14.3)	1 (12.5)	3 (13.6)
Past 3 months: overnight hospitalization	1 (7.1)	0 (0.0)	1 (4.5)

* Significant group differences, *p <* 0.05, ^a^ 1 KY participant did not complete medical utilization questions.

**Table 2:** Time-by-group interactions for primary and secondary outcome variables among study completers.

Variable	Baseline		Week 4		Week 8	
	KY	WLC	KY	WLC	KY	WLC
	M (SD) (*n* = 15)	M (SD) (*n* = 8)	M (SD) (*n* = 15)	M (SD) (*n* = 8)	M (SD) (*n* = 12) ^a^	M (SD) (*n* = 8)
Primary Outcomes						
Pain	5.87 (2.59)	5.12 (2.90)	4.93 (2.56)	4.25 (2.38)	4.50 (2.75)	3.75 (2.71)
Pain Interference	65.52 (9.43)	55.86 (7.89)	62.84 (11.85)	52.70 (7.86)	61.08 (11.68)	52.90 (8.28)
Fatigue	68.26 (9.77)	57.56 (7.92)	64.44 (8.70)	57.14 (7.93)	63.74 (6.46)	57.15 (9.44)
Global Health	1.86 (0.77)	2.25 (0.46)	2.00 (0.96)	2.25 (0.89)	2.58 (1.31)	2.62 (0.74)
Secondary Outcomes						
Multisystem Symptoms *	67.92 (25.81)	37.75 (11.47)	49.45 (26.73)	34.50 (13.50)	54.90 (26.48)	29.50 (16.32)
Depression (PROMIS)	59.70 (11.61)	51.85 (8.58)	56.44 (12.93)	47.97 (8.32)	56.74 (11.88)	48.71 (8.91)
Anxiety	62.64 (12.00)	53.98 (8.86)	56.96 (13.34)	51.10 (11.77)	58.78 (13.77)	52.17 (9.85)
Sleep Disturbance	58.98 (11.83)	56.10 (10.79)	60.45 (11.01)	55.01 (8.18)	55.95 (13.19)	54.14 (5.83)
Physical Functioning	38.24 (7.36)	42.66 (7.85)	38.88 (8.05)	39.80 (4.75)	37.41 (8.56)	41.26 (4.63)
Social Functioning	35.49 (8.41)	46.44 (8.72)	38.78 (6.95)	43.64 (6.28)	38.75 (6.85)	46.56 (5.86)
Depression (BDI)	22.92 (13.11)	13.63 (7.65)	18.5 (11.30)	13 (8.72)	16 (10.72)	11 (8.19)
Cognition *	26.77 (7.95)	39.38 (4.29)	30.22 (9.49)	37.73 (5.75)	31.66 (7.77)	37.61 (4.41)
Mindfulness	3.68 (1.08)	4.66 (0.85)	3.68 (1.18)	4.36 (1.05)	3.72 (1.34)	4.28 (0.78)

Note: Higher scores on all measures indicate more of the construct assessed. * Denotes variables where time-by-group interaction was significant, *p* < 0.05, ^a^ 2 participants did not complete week 8 measures. 1 participant’s week 8 measures were excluded due to starting a concomitant treatment mid-trial.

## Data Availability

The data presented in this study are openly available in Columbia University Academic Commons.

## Appendix A. Kundalini Yoga Daily Practice Protocol

### Appendix A.1. Part 1: Light Stretching

#### Duration: 8 Minutes

Participants completed dynamic stretching postures designed to be accessible regardless of fitness level and degree of physical pain. On the first day of the group therapy intervention, participants were trained to complete all dynamic stretches. They were also provided a copy of the form below detailing the dynamic stretching sequence:

1. *Standing:* March in place. Inhale and elevate your right knee while bending your right arm at the elbow. Then, exhale and lower your leg and arm simultaneously. Repeat this motion using your left leg and arm. Continue for one minute.
2. *Standing:* Inhale, raise your arms above your head and touch your palms together. Then, keeping your palms together and arms straight, exhale and bend towards the floor, hinging at the waist. Inhale again and return to the raised position. Continue for one minute.
3. *Sitting in a comfortable position with your legs crossed* **:* Shoulder lifts. Inhale and raise your shoulders. Exhale and lower your shoulders. Continue for one minute.
4. *Sitting in a comfortable position with your legs crossed* **:* Place your hands on your shoulders, ideally with your thumbs behind your shoulders, the rest of your fingers in front of your shoulders, and your elbows raised so that your arms are parallel to the floor. Inhale and twist your torso to the left, then exhale and twist your torso to the right. Continue for one minute. Finish with an inhale and exhale facing forward.
5. *Sitting in a comfortable position with your legs crossed* **:* Grab both ankles with your hands (or, if you are sitting in a chair, place your hands on your knees). Keep your elbows straight. Inhale and round your lumbar spine, then exhale and flex your lumbar spine. Continue for one minute.
6. *Sitting with legs stretched forward and spread apart in front of you* **:* Inhale and raise your arms above your head, then exhale and reach towards your right foot (or, if you are sitting in a chair, reach towards your right knee). Inhale and raise your arms above your head again, then exhale and reach towards the left foot (left knee if you are sitting in a chair). Continue for one minute.
7. *Sitting still in a comfortable position:* Breathe steadily. Remain attentive and mindful of your body. Focus inward for two minutes.

** If you are unable to sit in this position, sit in a chair with your feet flat on the floor.*

### Appendix A.2. Part 2: Directed Breathing

#### Duration: 8 Minutes

After completing the dynamic stretching sequence, participants performed the “breath of fire” central to Kundalini yoga practice. On the first day of the group therapy intervention, participants were trained in the breath of fire technique. Verbal guidance and support was provided during subsequent group meetings.

The breath of fire is a directed, rhythmic breathing pattern. Practitioners inhale and exhale through the nostrils, breathing from the diaphragm with the chest lifted and still, at an ideal rate of two breaths per second. Special attention is paid to make the breath equal in and out, with the exhale moving the diaphragm down and navel slightly inward. This balance of inhale and exhale assures smooth breaths with no hyperventilation or paradoxical breathing.

Participants were instructed to perform the breath of fire sitting comfortably in a chair or on the floor with legs crossed. Participants were encouraged to begin practicing the breath of fire at a rate that felt comfortable. Then, as their comfort with the technique improved, they increased their breathing rate towards the ideal of two breaths per second.

While performing the breath of fire, participants were instructed to perform a small rotation of the forearms at the elbow joint. In this rotation, the elbows were bent to form a 90° angle between the forearm and upper arm, with the forearms held perpendicular to the torso. Both wrists were held in a neutral position, and the tips of the thumbs touched the tips of the index fingers. The hands approached each other at the bottom of the rotation and diverged at the top. This movement was intended to be smooth and comfortable. However, if participants experienced too much pain to do this comfortably, the hand rotation could be done as a visualization.

After one and a half minutes of this synchronized breath of fire and forearm rotation, the tips of the thumbs were switched to touch the tips of the middle fingers for the following minute and a half. Then, sequentially, the tips of the thumbs were switched to touch the tips of the ring and pinky fingers, each for one and a half minutes.

When each fingertip had its turn engaging with the thumb, participants stopped the breath of fire and took one deep breath. Then, both hands were brought to the level of the heart, a few inches in front of the chest. There, the hands were cupped to bring the fingertips of one hand in contact with the same fingertips of the opposite hand. After one minute, the two cupped hands were separated to create a space of about 6 inches between the tips of the fingers. After another minute, participants took another deep breath.

### Appendix A.3. Part 3: Guided Meditation

#### Duration: 11 Minutes

After completing the breath of fire sequence, participants listened to a recorded narrative on focused awareness spoken over a calming musical background. Participants were given a CD or MP3 recording of the narrative to practice at home. The recording guided participants to envision a healing force or light, a positive energy field, circulating around and within and through themselves. When this force met areas of pain, participants were encouraged to visualize a transformation of this distress into healing.
